# Prevention of urinary tract infections in nursing homes: lack of evidence-based prescription?

**DOI:** 10.1186/1471-2318-11-69

**Published:** 2011-11-01

**Authors:** Jenny Bergman, Jan Schjøtt, Hege S Blix

**Affiliations:** 1 Regional medicines information and pharmacovigilance centre (RELIS Vest), Haukeland University Hospital, Bergen, Norway; 2 Section of Clinical Pharmacology, Laboratory of Clinical Biochemistry, Haukeland University Hospital, Bergen, Norway; 3 Section of Pharmacology, Institute of Medicine, University of Bergen, Bergen, Norway; 4 Department of Pharmacoepidemiology, Norwegian Institute of Public Health, Oslo, Norway

## Abstract

**Background:**

Urinary tract infections (UTIs, including upper and lower symptomatic) are the most common infections in nursing homes and prevention may reduce patient suffering, antibiotic use and resistance. The spectre of agents used in preventing UTIs in nursing homes is scarcely documented and the aim of this study was to explore which agents are prescribed for this purpose.

**Methods:**

We conducted a one-day, point-prevalence study in 44 Norwegian nursing homes during April-May 2006. Nursing home residents prescribed any agent for UTI prophylaxis were included. Information recorded was type of agent and dose, patient age and gender, together with nursing home characteristics. Appropriateness of prophylactic prescribing was evaluated with references to evidence in the literature and current national guidelines.

**Results:**

The study included 1473 residents. 18% (n = 269) of the residents had at least one agent recorded as prophylaxis of UTI, varying between 0-50% among the nursing homes. Methenamine was used by 48% of residents prescribed prophylaxis, vitamin C by 32%, and cranberry products by 10%. Estrogens were used by 30% but only one third was for vaginal administration. Trimethoprim and nitrofurantoin were used as prophylaxis by 5% and 4%, respectively.

**Conclusions:**

The agents frequently prescribed to prevent UTIs in Norwegian nursing homes lack documented efficacy including methenamine and vitamin C. Recommended agents like trimethoprim, nitrofurantoin and vaginal estrogens are infrequently used. We conclude that prescribing of prophylactic agents for UTIs in nursing homes is not evidence-based.

## Background

Urinary tract infections (UTIs) in the elderly include upper and lower symptomatic UTIs [1]. Asymptomatic bacteriuria is common and there is a wide consensus that this should not be treated. Guidelines have been developed for diagnostic criteria and treatment of UTIs [1-3]. In spite of this, studies show that a large proportion of antibacterial use in nursing homes may be inappropriate [4,5].

Nursing home residents frequently have infections and the prevalence is estimated to be 6-8%. UTIs accounts for around half of these infections and the estimates are similar irrespective of differences in study design [6-8]. In Norway, antimicrobial resistance among uropathogens has been relatively stable in the last decade [9]. Antimicrobial resistance pattern and health policy influences the national guidelines and pivmecillinam, trimethoprim and nitrofurantoin are the drugs of choice for treatment of acute symptomatic UTIs in Norway. Quinolones are only recommended in case of resistance or in upper UTI [3,10].

As a consequence of the high frequency of UTIs in nursing homes, several prevention strategies for recurrent UTIs are suggested. In addition to infection control programs and appropriate use of urinary catheters, use of prophylactic agents has been regarded as important. The use of prophylactic treatments for UTIs is commonly studied in subpopulations with risk factors like catheter use or urine reflux. These risk factors are prevalent in nursing homes, but little is known about UTI prophylaxis in these institutions. There is evidence that prophylaxis with low-dose antibiotics [11,12] and vaginal estrogens in postmenopausal women reduce the rate of UTIs [13]. In accordance with this, Norwegian guidelines recommend low dose of trimethoprim and nitrofurantoin as UTI prophylaxis in nursing homes residents. Vaginal administration of estrogens can also be tried [3]. Less proof is found for other prophylactic agents like methenamine and cranberries [14-16] although they are mentioned in the guidelines with a lower grade of evidence [3].

We could not find any studies looking at the prevalence and pattern of UTI prevention in nursing homes. The aim of this study was to explore which agents are used and the prescribing pattern for this purpose in nursing homes.

## Methods

### Study design

During April and May 2006, a one-day point-prevalence prescription study among all residents in 44 Norwegian nursing homes was carried out. The nursing homes were enrolled by members of the national network of consultant pharmacists. Data were collected by the use of two questionnaires and the consultant pharmacists were responsible for the data quality with the aid of nurses in the respective institutions. One questionnaire concerned the nursing home characteristics and demographics and the second questionnaire concerned the prescription of prophylactic agents for UTIs. Appropriateness of the prophylactic prescribing was evaluated with reference to evidence in the literature and current national guidelines [3]. The study was approved by the ethical committee and the Norwegian Social Science Service (NSD).

### Nursing homes

Nursing homes in Norway are community-based, not a part of hospitals, and populate 14% of the elderly aged 80 years and older. In 2006 there were 1003 nursing homes with 40 537 beds in Norway and 77% of the residents were ≥ 80 years of age (Statistics Norway http://www.ssb.no). Nursing homes provide 24 hrs nursing care and have own physicians employed, part or full time. The physicians are normally general practitioners (GPs) and specialisation in geriatrics is not compulsory. The nursing home physician has the medical responsibility for the patients in the nursing home and prescribes all drugs and other agents used for medicinal purposes in the medical record. Infection prevention and control specialists are not employed at nursing homes in Norway.

The data collected for each nursing home were number of residents, proportion of single rooms and level of care for each institution. Level of care was categorised as rehabilitation, long term somatic or dementia special care units when at least 70% of the beds fell into one of the categories. Otherwise it was called a mixed institution. Risk factors like catheter use and faecal incontinence were not recorded. Demographics included registration of gender and age.

### Prophylactic agents and antibiotics for therapy

All possible agents used for prevention of UTIs noted in the residents medical records were included, i.e. medicinal agents, vitamins and herbals, and they were recorded by name, daily dose and route of administration. Agents were classified according to The Anatomical Therapeutic Chemical (ATC) system [17]. Cranberry products represents a heterogeneous group of agents (including different doses of extracts, juices and capsules) but were coded together. The indication for UTI prophylaxis by the nursing home physician was accepted without further details of treatment criteria. Furthermore, length of treatment (according to the physician) was recorded in days. Prophylactic treatments are expected to be long-term and if no date of evaluation or withdrawal was assigned by the physician in the medical record, the treatments were recorded as continuous in the study.

### Statistical analysis

The data was analysed using SPSS 15.0 (SPSS Inc., Chicago IL). Descriptive statistics are shown as means with standard deviations (SD).

## Results

### Nursing home and resident characteristics

Forty-four nursing homes with a total of 1473 residents were included. The institutions were situated in central and northern parts of Norway in five out of 19 Norwegian counties. Data collection was performed by 11 consultant pharmacists. The average nursing home had 33 residents (SD = 18.0, range 8-86). 19% of the residents lived in dementia special care homes, 43% in long term somatic homes and 38% in mixed institutions. In 26 nursing homes (59%) all residents had single rooms; one nursing home had no single rooms while in the remaining 17 nursing homes the majority of the residents lived in single rooms. 81% of the residents prescribed prophylaxis were females and 61% were above 80 years of age.

### Prophylactic agents for UTIs

269 residents (18%) used at least one agent for UTI prophylaxis, Table 1. The proportion of residents with UTI prophylaxis varied between 0-50% among the nursing homes. Fifteen percent of the residents in homes for demented patients used prophylaxis for UTI, while 19% were prescribed UTI prophylaxis in long-term somatic and mixed institutions. However the pattern of prophylactic agents did not differ between the different types of institutions. Among residents using prophylactic agents, 74% had one agent prescribed, while 26% were prescribed two, three or four concomitant prophylactic agents, Table 1.

**Table 1:** Prophylaxis regimes for urinary tract infections in 1473 nursing home residents, number and proportions (%) of all residents and of residents with prophylaxis.

	Number	% of all residents	% of residents with prophylaxis
Residents given prophylaxis for UTIs	269	18%	
One agent	198	14%	74%
Two agents	63	4%	23%
Three or four agents	8	0.5%	3%
			
Agents used for prophylaxis			
Methenamine	130	8.8%	48%
Vitamin C	87	5.9%	32%
Estrogens	81	5.5%	30%
Cranberry	28	1.9%	10%
Trimethoprim	13	0.9%	5%
Nitrofurantoin	10	0.7%	4%

Methenamine was used by 48% of the residents prescribed prophylaxis, vitamin C by 32%, estrogens by 30% and cranberry products by 10%. In women receiving estrogens as prophylactic treatment, 25 (31%) had vaginal administered drugs and the others oral. Trimethoprim and nitrofurantoin were used as prophylaxis for UTIs in 9% of the residents, Table 1.

Dosages of prophylactic agents are shown in Table 2 and were within the recommended dosage range for prevention of UTIs as stated in the summary of product characteristics (SPC) for methenamine, estrogens, trimethoprim and nitrofurantoin. The prescribing of cranberry products appeared to follow suggested dosage by manufacturers in general, though recommendation sheets were not available for every commercial product used. The dosage of vitamin C varied between 60-2000 mg per day.

**Table 2:** Dosage of UTI prophylactic agents used in 1473 nursing home residents; Last columns: Norwegian guidelines on recommended UTI prophylactic treatments in nursing homes.

Dosage of UTI prophylactic agents used in 1473 nursing home residents			Norwegian guidelines: recommended UTI prophylaxis in nursing homes	
**Agents used**	**Mean (SD)**	**Dosage range**	**Dosage**	**Comments**

Methenamine	1.87 g (0.47)	1 - 2 g daily	1 g twice daily	Low grade documentation****
Vitamin C	490 mg (370)	60 - 2000 mg daily	-	Not recommended
Estrogens	-	0.5 - 2 g daily* 0.5 - 1.5 mg weekly**	-	Vaginal estrogens can be tried in recurrent UTIs
Cranberry	-	1 - 2 capsules daily***	-	Low grade documentation****
Trimethoprim	110 mg (26)	100 - 160 mg daily	100 mg daily	Consider local resistance pattern
Nitrofurantoin	70 mg (35)	50 - 150 mg daily	50 mg daily	Less effective with low or intermediate eGFR*****, pulmonal and kidney function must be assessed if long term treatment

* oral daily doses, ** vaginal weekly doses estriol (50 - 52 μg estradiol weekly), *** 2 residents received 4 ml mixture or 0.5 drinking glass of cranberry juice daily

**** Evidence grading system as commonly used in clinical practice Guidelines

***** Estimated glomerular filtration rate

## Discussion

The main finding of this study is that agents frequently prescribed to prevent UTIs in Norwegian nursing homes lack documented efficacy in the elderly. Recommended agents like trimethoprim, nitrofurantoin and vaginal estrogens are infrequently used. Instead we observed a high prevalence of methenamine, vitamin C, systemic estrogens and cranberry products. We conclude that prescribing of prophylactic agents for UTIs in nursing homes is not evidence-based according to the literature and current national guidelines.

Recurrent UTIs are common, especially in older women. Thus, high frequency of residents using prophylaxis in our study could be expected. However, the high variation in prevalence and the choices of prophylactic agents were surprising. One or several concomitant prophylactic agents were used by almost one fifth of the residents. At present we do not know which factors contribute to this high variation in prophylactic prevalence.

This and former studies have shown that the urinary antiseptic agent methenamine is frequently used in Norway and in Norwegian nursing homes, in contrast to most other countries in Europe [18,19]. In Norway, the use has even been increasing, and in 2010 methenamine represented 17% of antibacterials for systemic use, measured as share of DDDs among antiinfectives for systemic use (ATC-group J01) [20]. A Cochrane review of methenamine for preventing UTIs, found the overall quality of the studies to be poor. Few studies addressed long term use or the use in postmenopausal women or elderly in general. In the review it was concluded that methenamine may be effective for preventing UTI in patients without renal tract abnormalities, particularly when used for short-term prophylaxis [15]. In our study we found methenamine to be used frequently. The use appeared to be continuous which is in accordance with the SPC that does not state any limitations to treatment duration but in contrast with other documentation as summarized in the Cochrane review [15,21]. Current Norwegian guidelines include prophylactic use of methenamine as in patients without catheter, but point out the low grade of documentation for this agent [3].

Traditionally, cranberries have been used to prevent UTIs, but studies of efficacy have shown conflicting results. A recent Cochrane review concluded that cranberries could be effective, but that the evidence for the elderly still was inconclusive [16]. Another review did not recommend cranberry products for the prophylaxis of UTIs due to heterogeneity in study design and results, and a lack of consensus regarding both dosage regime and formulations. Interactions may be a problem in patients with polypharmacy, especially for concentrated cranberry products [22]. In addition, intolerance to cranberries probably represents a problem in the elderly and high withdrawal rates are reported in several studies [16,22]. In contrast to this, the withdrawal rates due to adverse reactions were the same comparing 500 mg cranberry extract with 100 mg trimethoprim in one study and with trimethoprim-sulfamethoxazole in another study. These studies included community dwelling women, age 45-93 years and 18 years to menopause respectively, and the relevance for nursing home residents is unclear [23,24].

Vaginal estrogens have been shown to decrease UTIs while systemic estrogens do not appear to have the same effect [13]. In our study only 31% (25 of 81) of the estrogens prescribed as UTI prophylaxis were for vaginal administration. This is troublesome because systemic estrogens have been associated with increased risk of cardiovascular disease, venous thromboembolic events and breast cancer [25]. Current Norwegian guidelines recommend vaginal estrogens to women with recurrent UTIs [3].

Trimethoprim and nitrofurantoin were the two antibiotics prescribed for prevention and used only by few of the nursing home patients. Long-term antibiotics are well documented to reduce the rate of UTIs but may be complicated by bacterial resistance and adverse drug reactions (ADRs) [11,12]. The prevalence of trimethoprim resistance in *Escherichia coli *isolates in Norway was 19% in 2007, being the drug with the highest prevalence of resistance in urinary tract isolates [9]. Nitrofurantoin was only resistant in 2.3% of the isolates [9]. However, nitrofurantoin should be used with caution in patients with renal impairment as reduced renal clearance increase the risk of ADRs and sufficient concentration in the urine depends on renal function. In addition, long term use of nitrofurantoin is associated with lung fibrosis and peripheral neuropathy [3,26]. As glomerular filtration rate decline by age, nursing home patients are at risk of a negative risk/benefit balance for the use of nitrofurantoin [27]. Local resistance pattern and individual renal function should therefore be considered before prescribing trimethoprim or nitrofurantoin for long-term use to nursing home residents.

Vitamin C has traditionally been regarded as effective in preventing recurrent UTIs. However, we could not find any studies showing that vitamin C is effective in preventing UTIs in the elderly. Norwegian guidelines do not recommend the use of vitamin C for UTI prophylaxis in nursing homes.

To summarize, our results show lack of evidence-based prescribing of prophylactic agents for UTIs in Norwegian nursing homes, according to evidence in the literature and current national guidelines. Low dose antibiotics and vaginal estrogens are at present recommended agents. However, they were only used by 18% of the residents prescribed prophylaxis in this study. The use of methenamine and cranberries were common, but the efficacy of these agents is not well documented. Systemic estrogens and vitamin C have no place in preventing UTIs and are to be considered as inappropriate as alternative and more evidence-based therapy exists. Prophylactic agents were prescribed continuously to residents, suggesting that their use were not regularly evaluated. The influence of catheter use and other risk factors are not known in our study, but an European surveillance study found the use of urinary catheter to be low (4,8%) in Norwegian nursing homes [28].

Generalisations from prevalence studies must be made carefully, but results can be useful to define quality improvement projects in institutions with less developed infection control systems than in hospitals. In previously published data from this study we showed prescribing of antibiotics for therapy to be in line with other studies from nursing homes [6,7,18]. We found all prescribing of UTI prophylaxis to be continuously which minimize the variation in results of this prevalence study. Interestingly, a recent Dutch study found a high occurrence of non-catheter related UTIs in nursing homes, perhaps due to frequent faecal incontinence among residents [29]. If this is true, prophylaxis should be focused on improving hygiene and providing incontinence materials rather than prescription of the agents found in our study. Notably, our study included a large number of nursing homes representing diversity in size and function. Thus, we believe the results from this study apply to Norwegian nursing homes in general, and that they also could be of international interest.

## Conclusions

This study showed a high prevalence and large variation in the use of UTI prophylactic agents in Norwegian nursing homes. The prescribing of agents to prevent UTIs was in most cases not in accordance with evidence in the literature or current national guidelines. The wide prescribing of agents that lack documentation of effectiveness is troublesome, and could represent an international problem as treatment of recurrent UTIs is a common challenge in the elderly. Thus, further studies are needed to clarify this prescribing behaviour and to evaluate possible benefits and disadvantages of prophylactic treatments in nursing home residents.

## Competing interests

The authors (JB, JS, HSB) declare that they have no competing interests. The authors are employed at their respective institutions. Otherwise, the study was carried out without financial support.

## Authors' contributions

All authors (JB, JS, HSB) have participated in the design and coordination, been involved in drafting the manuscript, critically reviewed the manuscript, read and approved the final manuscript.

## Pre-publication history

The pre-publication history for this paper can be accessed here:

http://www.biomedcentral.com/1471-2318/11/69/prepub
